# Functional capacity of individuals with brachial plexus injury

**DOI:** 10.3233/WOR-220414

**Published:** 2023-11-10

**Authors:** Tallie M.J. van der Laan, Sietke G. Postema, Corry K. van der Sluis, Michiel F. Reneman

**Affiliations:** Department of Rehabilitation Medicine, University Medical Center Groningen, University of Groningen, Groningen, The Netherlands

**Keywords:** Work capacity evaluation, upper extremity, musculoskeletal pain, disability, rehabilitation

## Abstract

**BACKGROUND:**

To enable (pain free) functioning, individuals with brachial plexus injury (BPI) may require a higher functional capacity compared to two-handed individuals, because the load on unaffected structures is greater.

**OBJECTIVE:**

This study compared the functional capacity of individuals with BPI and healthy controls and explored differences in the functional capacity of BPI-affected individuals with respect to: those with and without hand function; affected and unaffected sides; with and without musculoskeletal complaints (MSCs).

**METHODS:**

Six functional capacity tests adjusted for one-handed function were performed by 23 BPI-affected individuals and 20 healthy controls. Hand function was assessed through physical examination and the Dutch Musculoskeletal Questionnaire was used to assess MSCs.

**RESULTS:**

Individuals with BPI scored lower for the two-handed tests, compared with the controls (*p*≤0.01, effect size (r) ≤–0.41 for both tests). However, both groups performed similar in the one-handed tests. On average individuals with BPI met the physical demands to perform sedentary to light physical work. Among BPI-affected individuals, two-handed overhead lifting capacity was higher in those with hand function than in those without hand function (*p* = 0.02; *r* = 0.33). Functional capacity tended to be lower for the unaffected side than for the affected side (4 tests; *p*≤0.05, *r*≤–0.36). Test results of BPI-affected Individuals with and without MSCs were similar.

**CONCLUSION:**

Individuals with BPI demonstrated lower two-handed functional capacity than healthy controls. Effect sizes were medium. Capacity of their unaffected side was similar to the dominant side of controls. No association was found between MSCs and functional capacity.

## 1 Introduction

Returning to work poses a major challenge for adults with an acquired brachial plexus injury (BPI), with only 45% to 66% of these individuals resuming their previous work [1–4]. Of those who do, 31% have to adjust to or change jobs post-injury [2]. Most individuals (80%) with obstetric BPI report restrictions in the areas of work and education. Job and educational options for individuals with obstetric BPI are influenced by pain levels and limitations in their arm/hand function [5]. Studies have found that 50% to 86% of individuals with BPI experience pain, which is usually nerve or musculoskeletal in origin [2, 6–8]. Musculoskeletal complaints (MSCs) are defined as complaints localized in joints and muscles that are not caused by trauma or systemic disease [9]. MSCs have been reported in 49% of individuals with BPI, with the upper back, neck and unaffected upper limb being impacted significantly more often in these individuals compared with healthy controls [10]. Pain (neuropathic or MSCs) in individuals with BPI is associated with a lower quality of life [10, 11]. Individuals with BPI may be more prone to develop MSCs, given that limited functionality in an upper limb increasing the load on unaffected structures. This condition could increase exposure to known risk factors for MSCs, including forceful and repetitive movements, static muscle contractions, and working in awkward positions for prolonged periods[12].

Consequently, individuals with BPI may require higher functional capacity in the unaffected limb to compensate for the increased load on unaffected structures when performing daily and work-related tasks. Functional capacity is defined as “the highest probable level of functioning that a person can achieve in a given domain at a given moment within a standardized environment” [13], taking into consideration multiple biopsychosocial factors, including personal and environmental [13]. To the best of our knowledge there have been no studies till now on the functional capacity of individuals with BPI. Furthermore, nothing is known about the possible loss of functional capacity resulting from limited function in the BPI-affected arm.

The recently developed functional capacity evaluation one-handed (FCE-OH) can be applied to assess the functional capacity in individuals with BPI. The FCE-OH is a short-form functional capacity evaluation (FCE) adapted for individuals with upper limb absence and wearers of upper limb prostheses [14]. An FCE is used to evaluate the capacity of individuals to perform activities in order to make recommendations regarding their participation in work, while considering their body functions and structures, as well as environmental and personal factors and health status [14]. The assessment of the functional capacity can guide return-to-work recommendations, while potentially helping to prevent MSCs caused by overextension by matching functional capacity to physical job demands [15–17].

The primary aim of this study was to compare the functional capacity of individuals with BPI to that of healthy controls. Secondary aims were to explore differences in the functional capacity of BPI-affected individuals with respect to: those with and without hand function; affected and unaffected sides; with and without MSCs. In light of the finding of a previous study of one-handed individuals [14], we hypothesized that the two-handed functional capacity of individuals with BPI would be lower than that of healthy controls. Furthermore, we hypothesized that in one-handed tests the unaffected side of individuals with BPI would exhibit higher functional capacity relative to the dominant side of healthy controls, because of the adaptation required to carry extra loads.

## 2 Methods

### 2.1 Design

The study was designed as an observational case-control study.

### 2.2 Setting

**Fig. 1:**
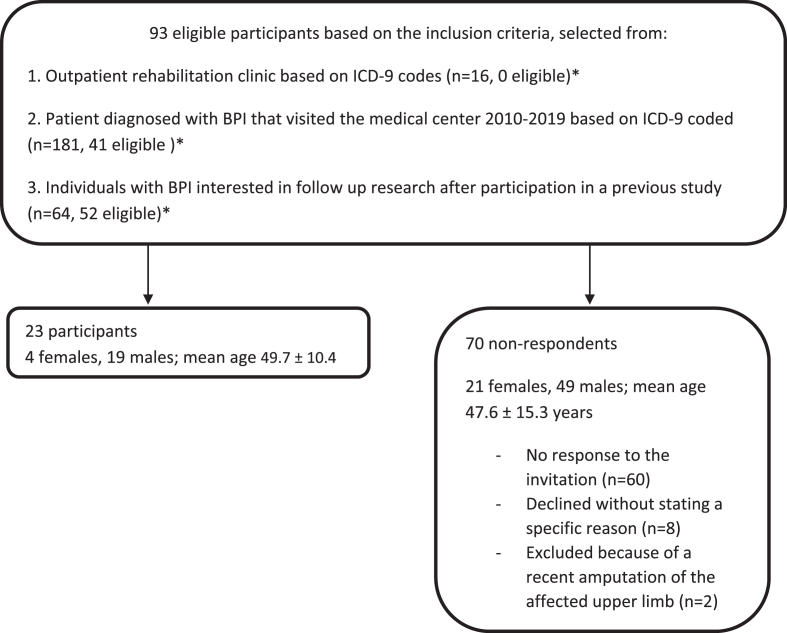
Participant recruitment. ^*^There were duplicates in the lists.

We recruited participants between February 2016 and May 2019. Individuals with BPI who had participated in a previous study and who had expressed interest in participating in subsequent research were invited to participate in the current study [10]. Furthermore, a search was performed in the database of the outpatient rehabilitation clinic of the university medical center and rehabilitation clinic using the International Classification of Disease (ICD-9) codes. All patients aged between 18 and 65 years, with a diagnosis of BPI who had visited the medical center from 2010 onwards were invited to participate in the study (Fig. 1). Data were collected from March 2016 to June 2019 at the local medical center.

### 2.3 Participants

Inclusion criteria for the study were adults aged between 18 and 65 years, who had sustained a BPI at least one year previously, were engaged in paid work, had a sufficient understanding of Dutch or English, and had normal function in the unaffected hand. Exclusion criteria were hypertension (blood pressure >160/100 mmHg at rest), serious pulmonary conditions, cardiac conditions, and other conditions that could cause unsafe situations during physical exertion. The seven-item physical activity readiness questionnaire (PAR-Q) was used to screen participants for serious health problems [18]. Participants responding “Yes” to one or more of the items were excluded.

The FCE-OH test results for individuals with BPI were compared to the results of the controls. The participants in the control group had previously participated in a pilot study to assess the functional capacity of individuals with major upper-limb defects [14]. Inclusion criteria for controls were: adults aged between 18 and 67 years, who had a good understanding of Dutch or English and normal hand function in both hands. Exclusion criteria for this group were the same as those for individuals with BPI.

### 2.4 Procedure

Participants made one visit to the university medical center. During this visit, they were asked to complete a questionnaire, followed by a physical examination of the upper limbs. Finally, they were asked to perform the FCE-OH tests.

#### 2.4.1 Questionnaire

Participants completed a questionnaire containing items about their age, sex, BPI (time since BPI, cause of BPI, nerve or muscle surgeries, and usage of the affected hand during routine daily activities). Pain was measured using the “Health 2” part of the Dutch Musculoskeletal Questionnaire (DMQ), which has good psychometric properties [19]. The DMQ was developed to measure the self-reported musculoskeletal workloads, risky working conditions, and MSCs. Within this questionnaire the “Health 2” component comprises of 11 items, for which participants are asked to rate their pain for each of the specified body parts using an eleven-point numeric rating scale (NRS; 0 no pain and –10 = extreme pain). MSCs were defined as pain or unpleasant sensations that are not caused by any acute trauma or systemic disease [9]. The DMQ does not differentiate between MSCs and neuropathic pain. Only complaints meeting the definition of a MSC in body parts other than the affected arm were classified as MSCs. An open question was added, to ascertain whether the participants had experienced other complaints (physical or mental) that could potentially influence their functional capacity.

#### 2.4.2 Hand function

Participants with BPI were divided into two groups; individuals with and without hand function. To the best of our knowledge, no functional scale currently exists for measuring remaining hand function in individuals with BPI. Classification of remaining hand function was therefore based on a physical examination and the following question: “Can you use the hand of the affected upper limb during activities of daily life?” This question was not validated, and was only used as an extra check in addition to the physical examination. The strength of hand and wrist muscles was measured using the Medical Research Council (MRC) scale, which has demonstrated good concurrent validity (Spearman’s rho 0.78) and satisfactory intra-rater and inter-rater reliability (kappa values 0.78–0.88) [20]. Appendix 1 provides an overview of the muscles that were tested. The Semmes-Weinstein monofilament test was used to detect sensation thresholds. This test is commonly used for individuals with peripheral nerve injuries, but has not been specifically validated for the use on BPI patients. The intra-rater reliability was good (kappa values 0.80–0.89) and the inter-rater reliability was satisfactory to good (kappa values 0.75–0.79; intra class correlation coefficient 0.97). The concurrent validity was satisfactory to good (Spearman’s rho 0.57–0.65) [21, 22]. The thumb, index finger, and little finger on the palmar side of the hand of each individual were each touched three times with Monofilament 2.83 (Appendix 2). If two of the three touches were sensed by the participant, no other Monofilament of a higher number was used, and the threshold detection was considered normal. If fewer than two of the three touches were sensed, the test was repeated with Monofilament 3.61 (the threshold for diminished light touch) and Monofilament 4.31 (the threshold for diminished protective sensation) [23]. Individuals demonstrating diminished sensation, a strength of less than MRC 3 in all tested hand and wrist muscles, and who responded “No” to the question of whether they could use the hand of the affected upper limb during activities of daily living were categorized in the “without hand function” category. All other individuals with BPI were categorized in the “with hand function” category. The cutoff point for hand function was based on the possibility moving the hand and wrist in all directions against gravity. This ability enabled participants to perform (some of) the one-handed FCE-tests and to use the hand during activities of daily living. Participants were unaware of the cut off point for hand function.

#### 2.4.3 FCE-OH

The FCE-OH consisted of six tests: two two-handed tests (overhead lifting two-handed and overhead working) and four one-handed tests (overhead lifting one-handed, repetitive reaching, fingertip-dexterity, and hand grip-strength) [14]. The objectives, instructions, and FCE outcomes for each test are described in Appendix 3. Participants performed the two-handed tests with two hands, wherever possible. If this was not possible because of limited strength in the shoulder and/or upper arm muscles, they performed the two-handed test with one hand. One-handed tests were performed twice: once with the unaffected side and once with the affected side, if the individual had sufficient strength in shoulder and/or upper arm muscles and had hand function (assessed as described above). If this was not the case, the test was performed only with the unaffected side. The tests were performed in a random order. During their performance, the participant’s heart rate was measured using a heart-rate monitor. The difference in heart rate before and after the test was calculated according to the following equation: [((heart rate post-test - heart rate pre-test)/heart rate pre-test)*100% ]. After each test was completed, the perceived load was measured using the Borg CR10 scale [16, 24]. The participants rated the perceived load using this scale, which ranges from zero to infinity. The scale has verbal anchors from zero (“no load at all”) to ten (“very high load”). The verbal anchor “absolute maximum” had no corresponding number. The FCE-OH was administered by one of three trained assessors. They were not blinded to results of the DMQ. Criteria for stopping the tests were as follows: (1) the participant expressed a desire to discontinue the test; (2) the participant’s heart rate exceeded the age-related maximum heart rate ((220-age)*85%); (3) the assessor detected an unsafe situation and (4) the test ended normally. No tests were commenced if the participant’s heart rate exceeded 70% of the age-related maximum.

#### 2.4.4 Ethics

This study was approved by the local medical ethics committee (METC 2013.038). All participants provided written informed consent before entering the study. All procedures followed were in accordance with the Helsinki Declaration of 1964, as revised in 2000.

### 2.5 Statistical analyses

For this study, a convenience sample size was used, in line with the study conducted by Postema et al. [14], in which the FCE-OH was pilot-tested on 20 patients with major upper-limb defects. SPSS for Windows (version 25.0.0; SPSS Advanced Statistics, Chicago, Illinois) was used for the data analysis.

Visual examination of QQ plots was used to check for normal distribution. Mann-Whitney U tests were performed to compare the FCE-OH results for individuals with BPI with those of healthy controls, as the data were not normally distributed. For one-handed tests, the results obtained for unaffected side were compared with those obtained for the controls dominant side. Test statistics (U) and effect sizes (r) were determined. Effect sizes were calculated by dividing the Z-score by the root of the study size [25]. The effect was considered small to medium sized if *r* < 0.3, medium sized if 0.3≤*r* < 0.5, and large if *r*≥0.5 [25]. Statistical significance was defined as a *p*-value ≤0.05.

Because of the exploratory nature of the study and the small subgroups descriptive statistics and Mann-Whitney U tests were used to explore differences in the FCE-OH test results of individuals with BPI with and without hand function, the affected and unaffected sides for individuals with BPI, and BPI-affected individuals with and without MSCs. Differences were considered significant if the *p*-value ≤0.10.

Cases with missing data in the FCE-OH test results, were only excluded from the analysis of the results of the respective FCE-OH tests. If there were missing data for the MSC items in the questionnaire or for the item on hand function, these cases were excluded for the analyses of the respective effects of MSCs or hand function on functional capacity.

## 3 Results

Of 93 eligible participants with BPI, 23 agreed to participate in this study (Fig. 1). Table 1 shows the characteristics of these participants and of the 20 controls. At least one year (minimum = 1.0 year, maximum = 50.0 years) had passed since the onset of BPI in all participants. All participants completed the FCE-OH tests, with no adverse effects observed or reported. None of the participants reported complaints other than pain (physical or mental), which could influence their functional capacity. Because of technical issues, the overhead working test was not performed by three BPI-affected individuals with hand function. Technical problems also resulted in missing data on heart rate differences (three individuals with BPI and one control).

**Table 1:** Characteristics of the participants

	Individuals with BPI			Controls
	*All individuals with BPI*	*Individuals with BPI, with MSC*	*Individuals with BPI, without MSC*
Number of participants	23	11	12	20
Gender male/female [*n*]	19/4	9/2	10/2	17/3
Age (years) [mean±SD]	49.7±10.4	52.6±7.9	46.8±11.8	45.8±12.4
Time since BPI (years) [mean±SD]	15.5±15.0	18.1±16.8	13.2±13.5
Causes of BPI [*n* (%)]
- Trauma	19 (82.6)	9 (81.8)	10 (83.3)
- Radiotherapy	2 (8.7)	1 (9.1)	1 (8.3)
- Obstetrical brachial plexus palsy	1 (4.3)	0 (0.0)	1 (8.3)
- Unknown	1 (4.3)	1 (9.1)	0 (0.0)
Side of BPI [*n* (%)]
- Right	12 (52.2)	5 (45.5)	7 (58.3)
- Left	11 (47.8)	6 (54.5)	5 (41.7)
BPI at the pre-existent dominant side [*n* (%)]	13 (56.5)	7 (63.6)	6 (50.0)
Surgery [*n* (%)]^a^	8 (34.8)	3 (27.3)	5 (41.7)
- Nerve surgery [*n*]	6	1	5
- Muscle transplantation [*n*]	1	0	1
- Surgery for fractures [*n*]	4	3	1
Function affected side
- No hand function [*n* (%)]	15 (65.2)	7 (63.6)	8 (66.7)
- No hand function [*n* (%)]	8 (34.8)	4 (36.4)	4 (33.3)
Active range of motion affected side [*n* (%)]
Normal	8 (34.7)	4 (36.4)	4 (33.3)
Diminished	11 (47.8)	6 (54.5)	5 (41.7)
No active motion possible	4 (17.4)	1 (9.1)	3 (25.0)
Sensation affected side [*n* (%)]
- Intact	1 (4.3)	1 (9.1)	0 (0.0)
- Diminished light touch	12 (52.2)	6 (54.5)	6 (50.0)
- Diminished protective sensation in one or more fingers	10 (43.5)	4 (36.4)	6 (50.0)
Pain at the day of testing [*n* (%)]	14 (60.9)			2 (10.0)
Musculoskeletal complaints [*n* (%)]	11 (47.8)			2 (10.0)
- 1 location		8		0
- 2 locations		2		1
- 3 locations		0		1
- 4 locations		1		0
Severity of Musculoskeletal complaints [*n*, mean NRS±SD]
- Neck		5, 3.4±1.5
- Unaffected shoulder		3, 3.3±1.2
- Back		4, 1.2±1.5
- Hip		2, 4.5±0.7
- Foot		1, 4.0

BPI = brachial plexus injury, MSC = musculoskeletal complaints, NRS = numeric pain rating scale, SD = standard deviation. ^a^Some participants had more than one surgery.

### 3.1 Functional capacity of individuals with BPI compared to controls

Six functional capacity tests were performed by 23 individuals with BPI and 20 healthy controls. The results of the tests are presented in Table 2. Individuals with BPI performed worse than the controls for two-handed overhead lifting (lifting a plastic receptacle), overhead working (participants manipulated nuts and bolts at crown height), and repetitive reaching (clicking buttons one wingspan apart). Effect sizes were medium to large. Perceived loads and increases in heart rate were similar in both groups. The results of the one-handed overhead lifting, fingertip dexterity and hand grip strength tests were also similar for individuals with BPI and controls.

**Table 2:** Functional capacity test results of individuals with BPI and controls

	Individuals with BPI (*n* = 23)	Controls (*n* = 20)	Test statistic (U)	*p*-value	Effect size (r)
Two-handed tests
*Overhead lifting test two-handed*					
Lifted weight in Kg	10.0 (8.0–17.0)	20.0 (17.0–24.0)	U = 71.5	***p*** < **0.00**	*r* = –0.59
Heart rate difference in %	36.1 (25.0–51.1)	41.9 (31.7–57.5)	U = 200.0	*p* = 0.47	*r* = –0.11
Perceived load CR 10	8.0 (5.0–10.0)	7.0 (5.2–7.8)	U = 184.0	*p* = 0.26	*r* = –0.17
*Overhead working test^*a*^*					
Time in seconds	183.5 (116.0–298.5)	312.0 (220.0–480.5)	U = 104.0	*p* = **0.01**	*r* = –0.41
Heart rate difference in %	16.0 (11.3–22.7)	15.1 (9.8–19.2)	U = 174.0	*p* = 0.67	*r* = –0.07
Perceived load CR 10	4.0 (3.0–5.0)	5.0 (3.3–6.0)	U = 150.0	*p* = 0.18	*r* = –0.23
One-handed tests
Overhead lifting test one-handed*					
Time in seconds	35.0 (31.8–47.0)	38.0 (34.5–43.0)	U = 208.0	*p* = 0.59	*r* = –0.08
Heart rate difference in %	18.9 (10.0–22.5)	12.0 (6.0–22.0)	U = 214.0	*p* = 0.70	*r* = –0.06
Perceived load CR 10	2.0 (1.0–3.0)	2.0 (1.0–3.0)	U = 209.5	*p* = 0.61	*r* = –0.08
Repetitive reaching test*					
Time in seconds	45.9 (41.3–57.7)	58.3 (53.7–65.5)	U = 132.5	*p* = **0.02**	*r* = –0.36
Heart rate difference in % ***^*b*^***	23.1 (13.9–31.7)	26.6 (21.9–38.0)	U = 154.0	*p* = 0.32	*r* = –0.16
Perceived load CR 10	2.0 (1.0–3.0)	2.0 (0.5–3.0)	U = 226.0	*p* = 0.92	*r* = –0.02
Fingertip dexterity test*					
Number of pins	14.3 (13.3–14.7)	14.0 (13.3–16.0)	U = 212.0	*p* = 0.66	*r* = 0.08
Perceived load CR 10	0.0 (0.0–1.0)	0.0 (0.0–1.0)	U = 205.0	*p* = 0.48	*r* = 0.11
Hand grip strength test*					
Hand grip strength in Kg	45.0 (37.7–48.7)	41.0 (35.8–49.8)	U = 216.5	*p* = 0.74	*r* = 0.05

All results are presented as median (m) and Inter Quartile Range (IQR). Abbreviations: BPI, brachial plexus injury; Kg, kilogram. ^a^Missing data: 3/23 individuals with BPI did not perform the test. ^b^Missing data: 3/23 individuals with BPI and 1/20 control. ^*^Individuals with BPI performed the test with the unaffected side, controls with the dominant side. Performance with the affected side/non-dominant side not presented in this table.

### 3.2 Functional capacity of individuals with BPI with and without hand function

The test results of 15 individuals with hand function (2 women and 13 men; mean age 49.9±10.9 years) were compared with those of 8 individuals without hand function (2 women and 6 men; mean age 49.1±9.9 years). Individuals with hand function performed better than those without hand function in the two-handed overhead lifting test, but they performed worse in the overhead working test (Table 3). Effect sizes were small to medium. No significant differences between these groups were observed in the one-handed FCE-OH test results.

**Table 3:** FCE-OH test results (a) of individuals with BPI with and without hand function, (b) of the affected and unaffected side of individuals with BPI and (c) of individuals with BPI with and without MSC

	Hand function (*n* = 15)	No hand function (*n* = 8)	Test statistics	Affected side^$^ (*n* = 15)	Unaffected side (*n* = 15)	Test statistics	MSC (*n* = 11)	No MSC (*n* = 12)	Test statistics
Two-handed tests
*Overhead lifting test two handed*									
Lifted weight in Kg	16 (9.0–18.0)	8.5 (6.5–9.0)	U = 22.5	NA	NA	NA	10.0 (8.0–17.0)	10.5 (6.8–17.5)	U = 62.5
			***p*** = **0.02**						*p* = 0.83
			*r* = 0.33						*r* = –0.10
*Overhead working test* ^#^									
Time in seconds	122.5 (106.8–276.8)	257.5 (161.0–308.8)	U = 26.0	NA	NA	NA	185 (122.5–380.0)	182.0 (115.0–299.0)	U = 43.0
			***p*** = **0.09**						*p* = 0.62
			*r* = 0.29						*r* = –0.16
One-handed tests
*Overhead lifting test one-handed*									
Time in seconds	35.0 (29.0–39.0)	42.0 (32.7–54.8)	U = 37.0	39.0 (31.0–50.0)	35.0 (29.0–39.0)	U = 5.0	37.8 (31.8–47.0)	35.0 (29.9–45.3)	U = 57.0
			*p* = 0.14			***p* <0.01**			*p* = 0.58
			*r* = –0.25			*r* = –0.51			*r* = –0.16
*Repetitive reaching test*									
Time in seconds	43.2 (13.7–57.7)	52.9 (42.4–59.7)	U = 48.0	49.5 (41.5–59.5)	43.2 (13.7–57.7)	U = 73.5	45.9 (37.8–57.7)	48.4 (41.8–60.0)	U = 57.0
			*p* = 0.44			***p*** = **0.05**			*p* = 0.58
			*r* = –0.20			*r* = –0.42			*r* = –0.17
*Fingertip dexterity test*									
Number of pins	14.3 (13.7–15.7)	14.5 (11.9–14.7)	U = 45.5	11.0 (10.0–14.0)	14.3 (13.7–15.7)	U = 7.5	14.3 (12.7–14.7)	14.5 (13.5–16.4)	U = 54.5
			*p* = 0.34			***p*** < **0.01**			*p* = 0.47
			*r* = 0.20			*r* = –0.43			*r* = –0.18
*Hand grip strength test*									
Hand grip strength Kg	45.7 (41.3–48.7)	43.8 (30.8–46.6)	U = 44.0	39.7 (18.7–47.7)	45.7 (41.3–48.7)	U = 25.5	43.0 (36.0–46.0)	46.5 (43.4–48.7)	U = 44.0
			*p* = 0.30			***p*** = **0.05**			*p* = 0.18
			*r* = –0.21			*r* = –0.36			*r* = –0.24

All results are presented as median (m) and Inter Quartile Range (IQR). Test statistics; Mann Whitney U (U), *p*-value (*p*) and effect size (r). Abbreviations: BPI, brachial plexus injury; MSC, musculoskeletal complaints; Kg, kilogram. ^#^Missing data overhead working test 3/15 with hand function, 2/11 with MSC, 1/12 without MSC.

### 3.3 One-handed tests: Functional capacity of the affected side compared with the unaffected side

The scores for the one-handed FCE-OH tests entailing the use of the affected side were significantly lower than those entailing the use of the unaffected side (Table 3). Effect sizes were medium to large.

### 3.4 Functional capacity and musculoskeletal complaints

No differences were observed in the characteristics of participants with and without MSCs (Table 1). Most individuals with MSCs experienced pain in the back, neck and unaffected shoulder. The occurrence of MSCs was similar in individuals with and without hand function and their pain intensity was low (Table 1). The results of all FCE tests were similar for participants with and without MSCs (Table 3).

## 4 Discussion

The two-handed functional capacity of individuals with BPI was significantly lower than that of healthy controls, whereas the one-handed functional capacity of the unaffected side of individuals with BPI was similar to that of the controls. This pattern was also observed among individuals with limited upper limb function caused by major upper limb defects [14]. In all the tests, BPI-affected individuals and controls demonstrated similar heart-rate differences and load perceptions, indicating that similar levels of physical effort were required in both groups during all the tests. These findings suggest that although two-handed functional capacity (and thus the amount of weight lifted) was lower for individuals with BPI, the same level of physical effort was exerted and the effect on heart rate was similar in both groups. This means that in assessment of the ability of individuals with BPI to work, it should not be assumed that these individuals expend less physical effort because they lift lower weights.

A comparison of the FCE-OH test results of individuals with BPI in our study with the FCE reference values for a healthy working population reveals that, on average, individuals with BPI are only able to meet the demands associated with sedentary to light physical work [26]. Job adaptations or changing jobs may thus be necessary. It is important to conduct assessments of functional capacity in relation to work demands for individuals, given the significant differences in the remaining function and functional capacity among individuals with BPI. Moreover, it is not known whether reference FCE values for the working population are applicable to individuals with BPI, as it is unclear whether physical requirements for certain types of work are the same for individuals with BPI and the general population. For example, individuals with BPI may deploy compensatory movement strategies, resulting in an extra load on bodily structures. Thus, higher upper limb work demands may be required for individuals with BPI compared with those of individuals with two well-functioning upper limbs for the same task. The reference values for the general population may therefore be too low. An understanding of how upper limb work demands for BPI-affected individuals relate to upper limb work demands in individuals with two well-functioning upper limbs, and how these work demands relate to FCE-OH test results, would improve recommendations on returning to work, thereby preventing work-related MSCs. Additionally, the validity of a comparison with healthy workers may also be questioned, as a narrow comparison restricted to workload may lead to underestimation of the total loading of individuals with BPI over a full day. The relative load for performing activities of daily living outside of work may be higher in patients with BPI, costs in terms of time and effort may be higher given difficulties in performing these activities [8, 27]. This situation may lead cumulatively to a higher overall daily loading of individuals with BPI.

The test results showed a lower one-handed functional capacity of the affected side compared with the unaffected side in individuals with BPI, which is probably attributable to BPI-induced loss of function. In a previous survey-based study, individuals with BPI reported using their unaffected side to compensate for the loss of function on the affected side [28], resulting in an increased load on the unaffected side. According to the dose-response theory, this extra load on the unaffected side could have two consequences [29]. The extra load could lead to adaptations in muscle tissue, possibly resulting in an increase in dose tolerance and capacity; it could also lead to muscle fatigue, MSCs and reduced capacity if the recovery time is insufficient.

Our results showed that the one-handed functional capacity of the unaffected side of individuals with BPI was similar to that of the dominant side of controls. This finding could be explained by the two possible consequences of the dose-response theory, depending on the activity level of individuals with BPI. BPI-affected individuals with reduced levels of activity had a correspondingly <s>a< /s>lower compensatory load on the unaffected side, resulting in little or no increase in functional capacity and a similar one-handed functional capacity compared with that of the controls. In case of similar activity levels of BPI-affected individuals and controls, and insufficient recovery time, the extra load could induce MSCs or muscle fatigue, which would result in a relatively lower capacity of the unaffected side. The latter may explain our observation of a similar one-handed functional capacity of BPI-affected individuals compared with that of the controls, as a higher capacity may be needed to fulfill the requirements of the participants’ daily tasks while avoiding muscle fatigue or MSCs. In order to differentiate between these two hypotheses, a follow-up study is needed to compare FCE-OH test results with daily and work activities and the presence of muscle fatigue in individuals with BPI and controls.

The finding of a higher two-handed overhead working capacity of individuals without hand function compared with that of individuals with hand function was significant. Individuals with hand function performed the test with both hands, while individuals without hand function performed the test with just the unaffected side. The lower overhead working capacity of individuals with hand function was probably caused by limited capacity of the affected side. This finding may have biased the comparison of the overhead working test results of individual with BPI and controls. In order control for this possible bias in future studies, all individuals (with BPI and controls) who performed the test two handed should also perform the test one handed. In this study we did not ask participants to perform both test conditions because a previous study [14] showed that the results will be influenced by fatigue. In that study the second performance of the overhead working test, independent of the test condition, was always lower [14].

Nearly half of the individuals with BPI in our study experienced MSCs. Although most individuals experienced MSCs in body parts that were subjected to stress during the FCE-OH tests (neck, back and unaffected shoulder), differences in the functional capacity of individuals with and without MSCs were small and non-significant. This finding is consistent with that of a study that investigated the functional capacity of individuals with major upper-limb deficiencies [14]. By contrast, Soer et al. [30] found that individuals with chronic MSCs (with no co-morbidities) had a lower functional capacity, relative to that of the general population. Pain intensity scores of individuals with BPI and those with major upper-limb deficiencies were low compared with the scores of individuals with chronic MSCs [14, 30]. Higher pain intensity scores were associated with lower FCE-OH tests scores [31], which could explain why no differences were found in the FCE-OH test results of individuals with and without MSCs. These findings indicate that the relationship between the presence of BPI, MSCs, and functional capacity remains uncertain, it should therefore be explored further within larger samples, preferably within a prospective study design. Additional insights into the relationship between these variables could help to improve the treatment of MSCs within this population.

### 4.1 Strengths

To the best of our knowledge the current study is the first to assess the functional capacity of individuals with BPI. It is also the first to explore differences in the functional capacity of the affected and unaffected side in individuals with BPI and between BPI-affected individuals with and without MSCs. We also included a control group for comparison purposes.

### 4.2 Limitations

Unlike most previous studies reported in the literature, we included individuals with adult and obstetric BPI in our study. Inclusion of persons with both types of BPI was not problematic in our study, as we aimed to assess functional capacity in BPI-affected individuals, irrespective of the cause of BPI. Individuals with obstetric BPI were probably better adapted to this condition compared with individuals with adult BPI, because they grew up with limited functionality of an upper limb. Whether or not this condition leads to a higher functional capacity is unknown. It would be interesting to compare the FCE-test results of individuals with obstetric BPI and those adult BPI who have the same remaining functionality in a follow-up study.

Most previous studies on BPI have classified plexus injuries according to the nerve roots involved and whether paralysis or paresis has occurred [2, 4, 11, 32]. However, the remaining functions can vary considerably among individuals with a paresis. Given the lack of a classification system based on remaining function we used a self-constructed classification based on remaining hand function in order to differentiate between one-handed and two-handed functioning individuals. The question (“Can you use the hand of the affected upper limb during activities of daily life?”) used in combination with the physical examination to differentiate between individuals with and without hand function was not validated. It is noteworthy that individuals in the “with hand function” category were able to perform (most) activities using both sides, but they could still experience limitations in hand function due to limited strength in the hand, wrist, upper arm and/or shoulder muscles. Individuals without hand function reported performing most activities with one hand, but some were able to use the (proximal) affected limb during some daily living activities depending on their remaining proximal strength.

Because of the small sample size relative to the scale of the differences and the heterogeneity of participants’ conditions, we were unable to draw generalizable conclusions concerning the associations between hand function, MSCs, and functional capacity in individuals with BPI. However, these results led to new hypotheses and could provide a direction for future studies and calculations of sample size. Because of the exploratory nature of this study and in order not to miss relevant hypotheses for future research, no corrections were made for multiple testing. However, the use of this approach increased the possibility of a type I error occurring and may have increased the chance of false positive results [33, 34].

The results of the FCE-OH tests may have been influenced by whether the individual’s dominant side was affected by BPI. Our post-hoc analysis to test this hypothesis revealed similar test results for individuals with BPI whose dominant side was or was not affected (see Appendix 4). The results were also potentially affected by selection bias, given that only 23 of the 93 prospective invited participants actually participated, although no differences in age or sex were observed between participants and non-participants.

Furthermore, the definition of MSCs applied in this study may have influenced the results. Because the DMQ does not differentiate between neuropathic pain and MSCs [19], pain felt on the affected side was classified as neuropathic pain and not classified as an MSC, with three individuals in the non-MSC group categorized as having pain in the affected side on the day of testing.

## 5 Conclusion

Consistent with our hypothesis, the two-handed functional capacity of individuals with BPI was found to be lower than that of healthy controls. The effect sizes were medium. The one-handed functional capacity of the unaffected side of individuals with BPI was similar to the capacity of the dominant side of controls and was not associated with hand function on the affected side. Presence of MSCs does not seem to be associated with functional capacity. More research into the relation between (daily and work) activities and functional capacity in individuals with BPI and controls is needed to shed further light on these results. Follow-up research may also help to uncover the implications of the FCE-OH for recommendations regarding work participation and provide insight into the causes of MSCs in individuals with BPI.

## Ethical approval

This study was approved by the Medical Ethics Committee of the University Medical Center Groningen, The Netherlands (METC 2013.038).

## Informed consent

All participants provided written informed consent before entering the study.

## Conflicts of interest

The authors declare that they have no conflict of interest.

## Supplementary materials

The appendices are available from https://dx.doi.org/10.3233/WOR-220414.

## References

[1] Holdenried M , Schenck T , Akpaloo J , Müller-Felber W , Holzbach T , Giunta R . Lebensqualität bei posttraumatischen Paresen des Plexus brachialis im Erwachsenenalter. Handchirurgie Mikrochirurgie Plast Chirurgie. 2013;45(04):229–34. 10.1055/s-0033-1353161 23970402

[2] Kretschmer T , Ihle S , Antoniadis G , Seidel JA , Heinen C , Börm W , Richter HP , König R . Patient Satisfaction and disablity after brachial plexus surgery. Neurosurgery. 2009;65(4):A189–96. 19927067 10.1227/01.NEU.0000335646.31980.33

[3] Haldane C , Frost G , Ogalo E , Bristol S , Doherty C , Berger M . A systematic review and meta-analysis of patient-reported outcomes following nerve transfer surgery for brachial plexus injury. PM and R. 2022. Available from: 10.1002/pmrj.12778. 35100499

[4] Gushikem A , Mendonca de Cardoso M , Cabral ALL , Mendes Barros CS , Isidro HBTM , Rodrigues Silva J , Barnetche Kauer J . Predictive factors for return to work or study and satisfaction in traumatic brachial plexus injury individuals undergoing rehabilitation: A retrospective follow-up study of 101 cases. J Hand Ther. 2021. . Available from: 10.1016/j.jht.2021.06.008. 34392998

[5] van der Holst M , Groot J , Steenbeek D , Pondaag W , Nelissen RGHH , Vliet Vlieland TPM . Participation restrictions among adolescents and adults with neonatal brachial plexus palsy: The patient perspective. Disabil Rehabil. 2018;40(26):3147–55. 28944700 10.1080/09638288.2017.1380717

[6] Ciaramitaro P , Mondelli M , Logullo F , Grimaldi S , Battiston B , Sard A , Scarinzi C , Migliaretti G , Faccani G , Cocito D . Traumatic peripheral nerve injuries: Epidemiological findings, neuropathic pain and quality of life in 158 patients. J Peripher Nerv Syst. 2010;15(2):120–7. 20626775 10.1111/j.1529-8027.2010.00260.x

[7] Santana MVB , Bina MT , Paz MG , Santos SN , Teixeira MJ , Raicher I , Martinis JV , Araujo Andrade DC , Baptista AF . High prevalence of neuropathic pain in the hand of patients with traumatic brachial plexus injury: A cross-sectional study. Arq Neuropsiquiatr. 2016;74(11):895–901. 27901254 10.1590/0004-282X20160149

[8] Partridge C , Edwards S . Obstetric brachial plexus palsy: Increasing disability and exacerbation of symptoms with age. Physiother Res Int. 2004;9(4):157–63. 15790253 10.1002/pri.319

[9] Huisstede BMA , Miedema HS , Verhagen AP , Koes BW , Verhaar JAN . Multidisciplinary consensus on the terminology and classification of complaints of the arm, neck and/or shoulder. Occup Environ Med. 2007;64(5):313–9. 17043078 10.1136/oem.2005.023861 PMC2092547

[10] van der Laan TMJ , Postema SG , van Bodegom J , Postema K , Dijkstra PU , van der Sluis CK . Prevalence and factors associated with musculoskeletal complaints and in individuals with brachial plexus injury: A cross-sectional study. Disabil Rehabil. 2022. Available from: 10.1080/09638288.2022.2117426. 36149019

[11] Estrella EP , Castillo-Carandang NT , Cordero CP , Juban NR . Quality of life of patients with traumatic brachial plexus injuries. Injury. 2021;52:855-61. Available from: 10.1016/j.injury.2020.11.074 33461770

[12] Gallagher S , Heberger JR . Examining the interaction of force and repetition on musculoskeletal disorder risk. Hum Factors J Hum Factors Ergon Soc. 2013;55(1):108–24. 10.1177/0018720812449648 PMC4495348 23516797

[13] Soer R , Van Der Schans CP , Groothoff JW , Geertzen JHB , Reneman MF . Towards consensus in operational definitions in functional capacity evaluation: A Delphi survey. J Occup Rehabil. 2008;18(4):389–400. 19011956 10.1007/s10926-008-9155-y

[14] Postema SG , Bongers RM , Reneman MF , Van Der Sluis CK . Functional capacity evaluation in upper limb reduction deficiency and amputation: Development and pilot testing. J Occup Rehabil. 2018;28(1):158–69. 28397018 10.1007/s10926-017-9703-4 PMC5820400

[15] Soer R , Gerrits EHJ , Reneman MF . Test-retest reliability of a WRULD functional capacity evaluation in healthy adults. Work. 2006;26(3):273–80. 16720967

[16] Reneman MF , Soer R , Gerrits EHJ . Basis for an FCE methodology for patients with work-related upper limb disorders. J Occup Rehabil. 2005;15(3):353–63. 16119226 10.1007/s10926-005-5942-x

[17] Lechner DE , Page JJ , Sheffield G . Predictive validity of a functional capacity evaluation: The physical work performance evaluation. Work. 2008;31:21–5. 18820417

[18] Thomas S , Reading RJS . Revision of the physical activity readiness questionnaire (PAR-Q). Can J Sport Sci. 1992;17(4):338–45. 1330274

[19] Hildebrandt VH , Bongers PM , van Dijk FJH , Kemper HCG , Dul J . Dutch musculoskeletal questionnaire: Description and basic. Ergonomics. 2010;44(12):1038–55. 10.1080/00140130110087437 11780727

[20] Paternostro-Sluga T , Grim-Stieger M , Posch M , Schuhfried O , Vacariu G , Mittermaier C , Bittner C , Fialka-Moser V . Reliability and validity of the Medical Research Council (MRC) scale and a modified scale for testing muscle strength in patients with radial palsy. J Rehabil Med. 2008;40(8):665–71. 19020701 10.2340/16501977-0235

[21] Suda M , Kawakami M , Okuyama K , Ishii R , Oshima O , Hijikata N , Nakamura T , Oka A , Kondo K , Liu M . Validity and reliability of the semmes-weinstein monofilament test and the thumb localizing test in patients with stroke. Front Neurol. 2021. Available from: 10.3389/fneur.2020.625917. PMC7873561 33584520

[22] Jerosch-Herhold C . Assessment of sensibility after nerve injury and repair: A systematic review of evidence for validity, reliability and responsiveness of tests. J of Hand Surgery. 2005;30B:252–64. 10.1016/j.jhsb.2004.12.006 15862365

[23] Bell-Krotoski JA , Fess EE , Figarola JH , Hiltz D . Threshold detection and Semmes-Weinstein monofilaments. J Hand Ther. 1995;8(2):155–62. 7550627 10.1016/s0894-1130(12)80314-0

[24] Borg G . Borg’s perceived exertion pain scales. Human Kinetics Publisher; 1998.

[25] Field A . Discovering Statistics Using SPSS. 4th ed. SAGE Publications; 2013.

[26] Soer R , van der Schans CP , Geertzen JH , Groothoff JW , Brouwer S , Dijkstra PU , Reneman MF . Normative values for a functional capacity evaluation. Arch Phys Med Rehabil. 2009;90(10):1785–94. 19801072 10.1016/j.apmr.2009.05.008

[27] Emmelot CH . Het plexus brachialis letsel: Een retrospectief onderzoek naar de functionele gevolgen. Van der Most Drukkerij te Heerde; 1994.

[28] Mancuso CA , Lee SK , Dy CJ , Landers ZA , Model Z , Wolfe SW . Compensation by the uninjured arm after brachial plexus injury. Hand. 2016;11(4):410–5. 28149206 10.1177/1558944715627635 PMC5256649

[29] Armstrong TJ , Buckle P , Fine JF , Hagberg M , Jonsson B , Kilbom A , Kuorinka IA , Silverstein BA , Sjogaard G , Viikari-Juntura ER . A conceptual model for work-related neck and upper-limb musculoskeletal disorders. Scand J Work Environ Heal. 1993;19(2):73–84. 10.5271/sjweh.1494 8316782

[30] Soer R , De Vries HJ , Brouwer S , Groothoff JW , Geertzen JH , Reneman MF . Do workers with chronic nonspecific musculoskeletal pain, with and without sick leave, have lower functional capacity compared with healthy workers? Arch Phys Med Rehabil. 2012;93(12):2216–22. 22772082 10.1016/j.apmr.2012.06.023

[31] Ansuategui Echeita J , Bethge M , van Holland BJ , Gross DP , Kool J , Oesch P , Trippolini MA , Chapman E , Cheng ASK , Sellars R , et al. Functional capacity evaluation in different societal contexts: Results of a multicountry study. J Occup Rehabil. 2019;29:222–36. 29802582 10.1007/s10926-018-9782-x PMC6510856

[32] Mancuso CA , Lee SK , Dy CJ , Landers ZA , Model Z , Wolfe SW . Expectations and limitations due to brachial plexus injury: A qualitative study. Hand. 2015;10(4):741–49. 26568734 10.1007/s11552-015-9761-z PMC4641103

[33] Armstrong RA . When to use the Bonferroni correction. Ophthalmic Physiol Opt. 2014;34(5):502–8. 24697967 10.1111/opo.12131

[34] Streiner DL , Norman GR . Correction for multiple testing: Is there a resolution? Chest. 2011;140(1):16–8. 21729890 10.1378/chest.11-0523

